# 3-Methyl-5-(3-phenoxy­phen­yl)cyclo­hex-2-enone

**DOI:** 10.1107/S1600536808012956

**Published:** 2008-05-10

**Authors:** R. T. Sabapathy Mohan, S. Kamatchi, M. Subramanyam, A. Thiruvalluvar, A. Linden

**Affiliations:** a Department of Chemistry, Annamalai University, Annamalai Nagar 608 002, Tamil Nadu, India; b PG Research Department of Physics, Rajah Serfoji Government College (Autonomous), Thanjavur 613 005, Tamil Nadu, India; c Institute of Organic Chemistry, University of Zürich, Winterthurerstrasse 190, CH-8057 Zürich, Switzerland

## Abstract

In the title mol­ecule, C_19_H_18_O_2_, the cyclo­hexene ring adopts an envelope conformation, with all substituents equatorial. The dihedral angle between the benzene and phenyl rings is 83.75 (16)°. No classical hydrogen bonds are found in the crystal structure.

## Related literature

For related literature, see: Pandiarajan *et al.* (2005 ▶).
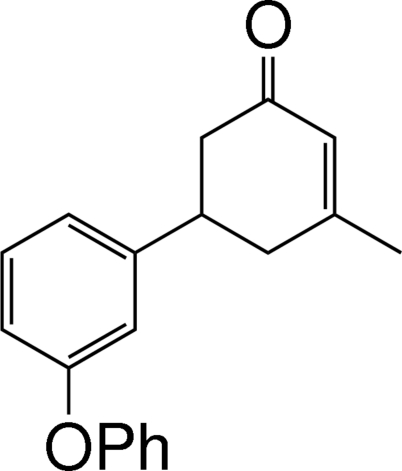


## Experimental

### 

#### Crystal data

C_19_H_18_O_2_
*M* *_r_* = 278.33
Orthorhombic, 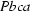

*a* = 9.6002 (3) Å
*b* = 17.1594 (7) Å
*c* = 17.6730 (5) Å
*V* = 2911.34 (17) Å^3^
*Z* = 8
Mo *K*α radiation
μ = 0.08 mm^−1^
*T* = 160 (1) K
0.20 × 0.15 × 0.08 mm

#### Data collection

Nonius KappaCCD area-detector diffractometer
Absorption correction: none
32821 measured reflections
2566 independent reflections
1708 reflections with *I* > 2σ(*I*)
*R*_int_ = 0.115

#### Refinement

*R*[*F* ^2^ > 2σ(*F* ^2^)] = 0.070
*wR*(*F* ^2^) = 0.198
*S* = 1.04
2566 reflections
191 parameters
H-atom parameters constrained
Δρ_max_ = 1.03 e Å^−3^
Δρ_min_ = −0.38 e Å^−3^

### 

Data collection: *COLLECT* (Nonius, 2000 ▶); cell refinement: *DENZO-SMN* (Otwinowski & Minor, 1997 ▶); data reduction: *DENZO-SMN* and *SCALEPACK* (Otwinowski & Minor, 1997 ▶); program(s) used to solve structure: *SHELXS97* (Sheldrick, 2008 ▶); program(s) used to refine structure: *SHELXL97* (Sheldrick, 2008 ▶); molecular graphics: *ORTEP-3* (Farrugia, 1997 ▶); software used to prepare material for publication: *PLATON* (Spek, 2003 ▶).

## Supplementary Material

Crystal structure: contains datablocks global, I. DOI: 10.1107/S1600536808012956/wn2258sup1.cif

Structure factors: contains datablocks I. DOI: 10.1107/S1600536808012956/wn2258Isup2.hkl

Additional supplementary materials:  crystallographic information; 3D view; checkCIF report

## Figures and Tables

**Fig. 1.**
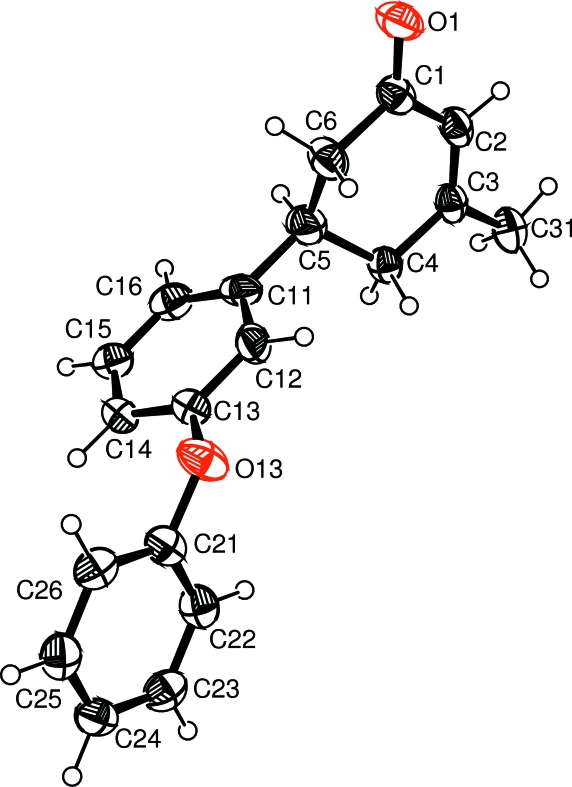
The molecular structure of the title compound, showing the atom-numbering scheme and displacement ellipsoids drawn at the 40% probability level. Hydrogen atoms are represented by spheres of arbitrary radius.

## supplementary crystallographic information

### Comment

The present X-ray diffraction study was undertaken to determine how the conformation of the system is affected by the substitution of a methyl group at position 3 and a phenoxyphenyl group at position 5 of the cyclohexenone ring. The molecular structure of the title compound, with atomic numbering scheme, is shown in Fig. 1. The cyclohexene ring adopts an envelope conformation, with all substituents equatorial. The dihedral angle between the benzene and phenyl rings is 83.75 (16)°. No classical hydrogen bonds are found in the crystal structure.

### Experimental

The title compound was prepared according to the general procedure reported by Pandiarajan *et al.* (2005). A mixture of 2,4-bis(ethoxycarbonyl)-5-hydroxy-5-methyl-3,3'-phenoxy phenylcyclohexanone (4.40 g, 0.01 mol) in glacial acetic acid (25 ml) and concentrated hydrochloric acid (50 ml) was refluxed for 12 h. After completion of the reaction, the reaction mixture was neutralized with aqueous ammonia and separated by using chloroform. The product was purified by column chromatography (benzene-EtOAc, 9.5:0.5 *v*/*v*). The yield of the isolated product was 2.08 g (75%).

### Refinement

H atoms were positioned geometrically and allowed to ride on their parent atoms, with C—H = 0.95–1.00 Å and *U*_iso_(H) = *xU*_eq_(carrier atom), where *x* = 1.5 for methyl and 1.2 for all other C atoms. The maximum residual electron density peak is located 1.08 Å from C5.

### Crystal data

**Table:**

C_19_H_18_O_2_	*D*_x_ = 1.270 Mg m^−^^3^
*M**_r_* = 278.33	Melting point: 478 K
Orthorhombic, *P**b**c**a*	Mo *K*α radiation λ = 0.71073 Å
Hall symbol: -P 2ac 2ab	Cell parameters from 2911 reflections
*a* = 9.6002 (3) Å	θ = 2.0–25.0º
*b* = 17.1594 (7) Å	µ = 0.08 mm^−^^1^
*c* = 17.6730 (5) Å	*T* = 160 (1) K
*V* = 2911.34 (17) Å^3^	Tablet, colourless
*Z* = 8	0.20 × 0.15 × 0.08 mm
*F*_000_ = 1184	

### Data collection

**Table:**

Nonius KappaCCD area-detector diffractometer	2566 independent reflections
Radiation source: Nonius FR590 sealed tube generator	1708 reflections with *I* > 2σ(*I*)
Monochromator: horizontally mounted graphite crystal	*R*_int_ = 0.115
Detector resolution: 9 pixels mm^-1^	θ_max_ = 25.1º
*T* = 160(1) K	θ_min_ = 2.4º
ω scans with κ offsets	*h* = −11→11
Absorption correction: none	*k* = −20→20
32821 measured reflections	*l* = −21→21

### Refinement

**Table:**

Refinement on *F*^2^	Secondary atom site location: difference Fourier map
Least-squares matrix: full	Hydrogen site location: inferred from neighbouring sites
*R*[*F*^2^ > 2σ(*F*^2^)] = 0.070	H-atom parameters constrained
*wR*(*F*^2^) = 0.198	*w* = 1/[σ^2^(*F*_o_^2^) + (0.0847*P*)^2^ + 2.668*P*] where *P* = (*F*_o_^2^ + 2*F*_c_^2^)/3
*S* = 1.04	(Δ/σ)_max_ < 0.001
2566 reflections	Δρ_max_ = 1.04 e Å^−^^3^
191 parameters	Δρ_min_ = −0.38 e Å^−^^3^
Primary atom site location: structure-invariant direct methods	Extinction correction: none

### Special details

**Table:**

Experimental. Solvent used: ? Cooling Device: Oxford Cryosystems Cryostream 700 Crystal mount: glued on a glass fibre Mosaicity (°.): 0.560 (2) Frames collected: 270 Seconds exposure per frame: 114 Degrees rotation per frame: 1.2 Crystal-Detector distance (mm): 30.0
Geometry. Bond distances, angles *etc*. have been calculated using the rounded fractional coordinates. All su's are estimated from the variances of the (full) variance-covariance matrix. The cell e.s.d.'s are taken into account in the estimation of distances, angles and torsion angles
Refinement. Refinement of *F*^2^ against ALL reflections. The weighted *R*-factor *wR* and goodness of fit *S* are based on *F*^2^, conventional *R*-factors *R* are based on *F*, with *F* set to zero for negative *F*^2^. The threshold expression of *F*^2^ > σ(*F*^2^) is used only for calculating *R*-factors(gt) *etc*. and is not relevant to the choice of reflections for refinement. *R*-factors based on *F*^2^ are statistically about twice as large as those based on *F*, and *R*- factors based on ALL data will be even larger.

### Fractional atomic coordinates and isotropic or equivalent isotropic displacement parameters (Å^2^)

**Table:**

	*x*	*y*	*z*	*U*_iso_*/*U*_eq_	
O1	−0.0247 (3)	−0.18365 (15)	0.28956 (14)	0.0629 (9)	
O13	0.5599 (2)	0.05049 (13)	0.11517 (15)	0.0591 (9)	
C1	−0.0118 (3)	−0.14143 (19)	0.2341 (2)	0.0455 (11)	
C2	−0.1041 (3)	−0.14696 (19)	0.16869 (19)	0.0451 (11)	
C3	−0.0947 (3)	−0.09996 (18)	0.10876 (19)	0.0413 (10)	
C4	0.0161 (3)	−0.03832 (18)	0.10374 (17)	0.0395 (10)	
C5	0.0737 (4)	−0.0131 (2)	0.1805 (2)	0.0567 (12)	
C6	0.1045 (4)	−0.0818 (2)	0.2293 (2)	0.0550 (12)	
C11	0.1921 (3)	0.0452 (2)	0.17330 (17)	0.0493 (13)	
C12	0.3246 (4)	0.0212 (2)	0.14640 (18)	0.0514 (11)	
C13	0.4298 (3)	0.07684 (19)	0.13841 (17)	0.0412 (10)	
C14	0.4052 (3)	0.15345 (18)	0.15702 (17)	0.0399 (10)	
C15	0.2749 (3)	0.1745 (2)	0.18305 (18)	0.0478 (11)	
C16	0.1706 (4)	0.1217 (2)	0.19066 (18)	0.0523 (14)	
C21	0.6445 (3)	0.10391 (19)	0.07643 (19)	0.0450 (11)	
C22	0.6069 (3)	0.1299 (2)	0.0053 (2)	0.0490 (11)	
C23	0.6939 (4)	0.1795 (2)	−0.0331 (2)	0.0512 (11)	
C24	0.8177 (4)	0.20337 (19)	−0.0009 (2)	0.0485 (11)	
C25	0.8541 (4)	0.1771 (2)	0.0703 (2)	0.0517 (11)	
C26	0.7668 (3)	0.1271 (2)	0.10933 (19)	0.0472 (11)	
C31	−0.1933 (3)	−0.1052 (2)	0.0437 (2)	0.0547 (12)	
H2	−0.17441	−0.18594	0.16881	0.0541*	
H4A	0.09381	−0.05832	0.07242	0.0474*	
H4B	−0.02296	0.00786	0.07774	0.0474*	
H5	−0.00388	0.01545	0.20614	0.0677*	
H6A	0.18905	−0.10791	0.20966	0.0662*	
H6B	0.12561	−0.06310	0.28098	0.0662*	
H12	0.34146	−0.03181	0.13401	0.0619*	
H14	0.47676	0.19132	0.15203	0.0479*	
H15	0.25785	0.22737	0.19600	0.0576*	
H16	0.08181	0.13819	0.20821	0.0627*	
H22	0.52162	0.11346	−0.01684	0.0588*	
H23	0.66872	0.19757	−0.08207	0.0616*	
H24	0.87765	0.23777	−0.02755	0.0582*	
H25	0.93940	0.19332	0.09252	0.0622*	
H26	0.79133	0.10911	0.15838	0.0566*	
H31A	−0.26101	−0.14680	0.05316	0.0820*	
H31B	−0.24236	−0.05547	0.03789	0.0820*	
H31C	−0.14127	−0.11674	−0.00267	0.0820*	

### Atomic displacement parameters (Å^2^)

**Table:**

	*U*^11^	*U*^22^	*U*^33^	*U*^12^	*U*^13^	*U*^23^
O1	0.0680 (17)	0.0558 (16)	0.0649 (16)	−0.0136 (13)	0.0041 (13)	0.0168 (14)
O13	0.0528 (15)	0.0379 (14)	0.0866 (18)	0.0057 (12)	0.0163 (13)	0.0127 (12)
C1	0.0423 (18)	0.0383 (18)	0.056 (2)	0.0021 (15)	0.0055 (15)	0.0018 (17)
C2	0.0354 (17)	0.0379 (19)	0.062 (2)	−0.0055 (14)	0.0070 (15)	−0.0097 (16)
C3	0.0336 (16)	0.0369 (18)	0.0533 (19)	0.0012 (14)	0.0011 (14)	−0.0100 (15)
C4	0.0351 (16)	0.0394 (18)	0.0440 (17)	−0.0002 (14)	−0.0006 (13)	−0.0026 (14)
C5	0.054 (2)	0.063 (2)	0.053 (2)	−0.0137 (19)	−0.0044 (17)	0.0033 (18)
C6	0.060 (2)	0.044 (2)	0.061 (2)	−0.0104 (17)	−0.0137 (18)	0.0118 (17)
C11	0.0363 (18)	0.077 (3)	0.0347 (17)	−0.0184 (17)	−0.0048 (14)	0.0115 (17)
C12	0.072 (2)	0.0363 (19)	0.0459 (19)	−0.0121 (18)	−0.0090 (17)	0.0034 (15)
C13	0.0388 (17)	0.0402 (19)	0.0446 (18)	−0.0023 (15)	0.0004 (14)	0.0055 (14)
C14	0.0372 (17)	0.0372 (19)	0.0452 (18)	−0.0040 (14)	−0.0012 (14)	0.0045 (14)
C15	0.047 (2)	0.055 (2)	0.0415 (18)	0.0008 (17)	0.0018 (15)	0.0035 (16)
C16	0.048 (2)	0.067 (3)	0.0420 (19)	0.0032 (19)	0.0000 (15)	0.0037 (17)
C21	0.0392 (17)	0.0357 (18)	0.060 (2)	0.0048 (15)	0.0081 (16)	0.0010 (16)
C22	0.0410 (18)	0.047 (2)	0.059 (2)	0.0015 (16)	−0.0023 (16)	−0.0042 (17)
C23	0.056 (2)	0.050 (2)	0.0476 (19)	0.0128 (18)	0.0041 (17)	−0.0002 (16)
C24	0.050 (2)	0.0376 (19)	0.058 (2)	0.0036 (15)	0.0159 (17)	0.0008 (16)
C25	0.0421 (19)	0.053 (2)	0.060 (2)	0.0040 (17)	−0.0005 (17)	−0.0115 (18)
C26	0.0466 (19)	0.049 (2)	0.0461 (19)	0.0116 (16)	0.0019 (16)	0.0005 (16)
C31	0.050 (2)	0.048 (2)	0.066 (2)	−0.0049 (17)	−0.0103 (17)	−0.0078 (18)

### Geometric parameters (Å, °)

**Table:**

O1—C1	1.225 (4)	C24—C25	1.382 (5)
O13—C13	1.390 (4)	C25—C26	1.384 (5)
O13—C21	1.403 (4)	C2—H2	0.9500
C1—C2	1.460 (5)	C4—H4A	0.9900
C1—C6	1.517 (5)	C4—H4B	0.9900
C2—C3	1.334 (5)	C5—H5	1.0000
C3—C4	1.503 (4)	C6—H6A	0.9900
C3—C31	1.492 (5)	C6—H6B	0.9900
C4—C5	1.528 (5)	C12—H12	0.9500
C5—C6	1.490 (5)	C14—H14	0.9500
C5—C11	1.520 (5)	C15—H15	0.9500
C11—C12	1.419 (5)	C16—H16	0.9500
C11—C16	1.364 (5)	C22—H22	0.9500
C12—C13	1.397 (5)	C23—H23	0.9500
C13—C14	1.376 (4)	C24—H24	0.9500
C14—C15	1.381 (4)	C25—H25	0.9500
C15—C16	1.357 (5)	C26—H26	0.9500
C21—C22	1.382 (5)	C31—H31A	0.9800
C21—C26	1.369 (4)	C31—H31B	0.9800
C22—C23	1.372 (5)	C31—H31C	0.9800
C23—C24	1.380 (5)		
			
O1···H23^i^	2.6700	H4A···C23^x^	2.9900
O1···H15^ii^	2.7200	H4A···C24^x^	2.9200
O13···H6B^iii^	2.7500	H4B···C26^viii^	2.9300
C2···C6^iv^	3.511 (5)	H4B···H31B	2.4700
C6···C2^iii^	3.511 (5)	H5···C2	3.0200
C14···C22	3.332 (4)	H5···H16	2.2600
C22···C14	3.332 (4)	H5···C12^iv^	3.0800
C24···C31^v^	3.583 (5)	H5···C13^iv^	3.0100
C31···C24^vi^	3.583 (5)	H6A···C12	2.8000
C1···H6A^iv^	3.0900	H6A···H12	2.3700
C2···H6A^iv^	3.0000	H6A···C1^iii^	3.0900
C2···H6B^iv^	3.1000	H6A···C2^iii^	3.0000
C2···H5	3.0200	H6B···O13^iv^	2.7500
C2···H14^vi^	3.0500	H6B···C2^iii^	3.1000
C6···H12	2.9600	H12···C6	2.9600
C12···H5^iii^	3.0800	H12···H6A	2.3700
C12···H4A	2.9100	H12···C22^x^	3.0200
C12···H6A	2.8000	H14···C21	2.5700
C13···H22	2.9500	H14···C22	3.0700
C13···H5^iii^	3.0100	H14···C26	3.0900
C14···H24^vii^	2.9600	H14···C2^v^	3.0500
C14···H16^iii^	2.9400	H15···H23^vii^	2.5400
C15···H23^vii^	3.0100	H15···O1^xi^	2.7200
C15···H26^iv^	3.0200	H16···H5	2.2600
C16···H25^viii^	3.0700	H16···C14^iv^	2.9400
C16···H26^iv^	2.9200	H22···C13	2.9500
C21···H31B^ix^	3.0200	H23···O1^xii^	2.6700
C21···H14	2.5700	H23···C15^xiii^	3.0100
C22···H14	3.0700	H23···H15^xiii^	2.5400
C22···H12^x^	3.0200	H24···C14^xiii^	2.9600
C23···H4A^x^	2.9900	H25···C16^ix^	3.0700
C24···H4A^x^	2.9200	H26···C15^iii^	3.0200
C24···H31A^v^	2.8000	H26···C16^iii^	2.9200
C26···H4B^ix^	2.9300	H31A···H2	2.3100
C26···H14	3.0900	H31A···C24^vi^	2.8000
H2···H31A	2.3100	H31B···C21^viii^	3.0200
H4A···C12	2.9100	H31B···H4B	2.4700
			
C13—O13—C21	116.9 (2)	C5—C4—H4A	109.00
O1—C1—C2	122.3 (3)	C5—C4—H4B	109.00
O1—C1—C6	121.2 (3)	H4A—C4—H4B	108.00
C2—C1—C6	116.5 (3)	C4—C5—H5	106.00
C1—C2—C3	123.3 (3)	C6—C5—H5	106.00
C2—C3—C4	121.3 (3)	C11—C5—H5	106.00
C2—C3—C31	122.2 (3)	C1—C6—H6A	109.00
C4—C3—C31	116.5 (3)	C1—C6—H6B	109.00
C3—C4—C5	113.8 (3)	C5—C6—H6A	109.00
C4—C5—C6	111.2 (3)	C5—C6—H6B	109.00
C4—C5—C11	112.5 (3)	H6A—C6—H6B	108.00
C6—C5—C11	114.9 (3)	C11—C12—H12	121.00
C1—C6—C5	114.8 (3)	C13—C12—H12	121.00
C5—C11—C12	120.5 (3)	C13—C14—H14	120.00
C5—C11—C16	120.1 (3)	C15—C14—H14	120.00
C12—C11—C16	119.4 (3)	C14—C15—H15	119.00
C11—C12—C13	118.9 (3)	C16—C15—H15	119.00
O13—C13—C12	117.2 (3)	C11—C16—H16	120.00
O13—C13—C14	122.4 (3)	C15—C16—H16	120.00
C12—C13—C14	120.3 (3)	C21—C22—H22	120.00
C13—C14—C15	119.0 (3)	C23—C22—H22	120.00
C14—C15—C16	121.8 (3)	C22—C23—H23	120.00
C11—C16—C15	120.6 (3)	C24—C23—H23	120.00
O13—C21—C22	120.2 (3)	C23—C24—H24	120.00
O13—C21—C26	118.6 (3)	C25—C24—H24	120.00
C22—C21—C26	121.1 (3)	C24—C25—H25	120.00
C21—C22—C23	119.4 (3)	C26—C25—H25	120.00
C22—C23—C24	120.3 (3)	C21—C26—H26	120.00
C23—C24—C25	119.8 (3)	C25—C26—H26	120.00
C24—C25—C26	120.2 (3)	C3—C31—H31A	109.00
C21—C26—C25	119.2 (3)	C3—C31—H31B	109.00
C1—C2—H2	118.00	C3—C31—H31C	109.00
C3—C2—H2	118.00	H31A—C31—H31B	109.00
C3—C4—H4A	109.00	H31A—C31—H31C	109.00
C3—C4—H4B	109.00	H31B—C31—H31C	109.00
			
C21—O13—C13—C12	153.1 (3)	C6—C5—C11—C16	−125.0 (3)
C21—O13—C13—C14	−30.3 (4)	C5—C11—C12—C13	178.1 (3)
C13—O13—C21—C22	−67.2 (4)	C16—C11—C12—C13	0.0 (5)
C13—O13—C21—C26	115.2 (3)	C5—C11—C16—C15	−178.7 (3)
O1—C1—C2—C3	177.8 (3)	C12—C11—C16—C15	−0.6 (5)
C6—C1—C2—C3	−3.8 (5)	C11—C12—C13—O13	177.2 (3)
O1—C1—C6—C5	−153.3 (3)	C11—C12—C13—C14	0.6 (5)
C2—C1—C6—C5	28.3 (4)	O13—C13—C14—C15	−177.0 (3)
C1—C2—C3—C4	1.6 (5)	C12—C13—C14—C15	−0.5 (5)
C1—C2—C3—C31	−178.0 (3)	C13—C14—C15—C16	−0.1 (5)
C2—C3—C4—C5	−23.1 (4)	C14—C15—C16—C11	0.7 (5)
C31—C3—C4—C5	156.5 (3)	O13—C21—C22—C23	−177.2 (3)
C3—C4—C5—C6	45.7 (4)	C26—C21—C22—C23	0.3 (5)
C3—C4—C5—C11	176.2 (3)	O13—C21—C26—C25	177.2 (3)
C4—C5—C6—C1	−48.6 (4)	C22—C21—C26—C25	−0.3 (5)
C11—C5—C6—C1	−177.8 (3)	C21—C22—C23—C24	−0.2 (5)
C4—C5—C11—C12	−71.7 (4)	C22—C23—C24—C25	0.1 (5)
C4—C5—C11—C16	106.4 (3)	C23—C24—C25—C26	−0.1 (5)
C6—C5—C11—C12	56.9 (4)	C24—C25—C26—C21	0.2 (5)

Symmetry codes: (i) −*x*+1/2, −*y*, *z*+1/2; (ii) −*x*, *y*−1/2, −*z*+1/2; (iii) *x*+1/2, *y*, −*z*+1/2; (iv) *x*−1/2, *y*, −*z*+1/2; (v) −*x*+1/2, *y*+1/2, *z*; (vi) −*x*+1/2, *y*−1/2, *z*; (vii) *x*−1/2, −*y*+1/2, −*z*; (viii) *x*−1, *y*, *z*; (ix) *x*+1, *y*, *z*; (x) −*x*+1, −*y*, −*z*; (xi) −*x*, *y*+1/2, −*z*+1/2; (xii) −*x*+1/2, −*y*, *z*−1/2; (xiii) *x*+1/2, −*y*+1/2, −*z*.
